# In Search of the Elusive North: Evolutionary History of the Arctic Fox (*Vulpes lagopus*) in the Palearctic from the Late Pleistocene to the Recent Inferred from Mitogenomic Data

**DOI:** 10.3390/biology12121517

**Published:** 2023-12-12

**Authors:** Valentina A. Panitsina, Semyon Yu. Bodrov, Eugenia S. Boulygina, Natalia V. Slobodova, Pavel A. Kosintsev, Natalia I. Abramson

**Affiliations:** 1 Zoological Institute, Russian Academy of Sciences, 199034 Saint-Petersburg, Russia; valentina.panitsina@zin.ru (V.A.P.); semyon.bodrov@zin.ru (S.Y.B.); 2 National Research Center “Kurchatov Institute”, 123182 Moscow, Russia; eugenia.bulygina@gmail.com; 3 Faculty of Biology and Biotechnology, HSE University, 101000 Moscow, Russia; 4 Institute of Plant and Animal Ecology, Ural Branch, Russian Academy of Sciences, 620144 Yekaterinburg, Russia

**Keywords:** ancient DNA, arctic fox, mitochondrial genome, the Urals, Pleistocene, Holocene

## Abstract

**Simple Summary:**

Global warming at the border of Late Pleistocene-Holocene, around ten thousand years ago caused a dramatic rearrangement of habitats in the Northern Hemisphere. Populations of cold-adapted megafauna species, which were spread over large areas of Eurasia, did not survive it. At the same time, small representatives of this mammoth fauna complex survived, including lemmings and arctic fox, but greatly reduced their distribution northward. However, it is uncertain whether species survived by habitat tracking the elusive tundra, or if they came from other places where they survived warming, and local populations died out without leaving descendants. To answer this, we studied ancient DNA from new fossil remains of arctic foxes from caves in the northern and polar Urals. The data received do not show any connectivity between ancient and modern individuals, supporting the hypothesis of local extinction of arctic fox in the region rather than the tracking habitat hypothesis. These findings are important in light of global climate warming expectations. It is predicted that the most severe effects are expected to occur in high-latitude biomes and the results obtained must be kept in mind when planning conservation policy measures.

**Abstract:**

Despite the high level of interest, the population history of arctic foxes during the Late Pleistocene and Holocene remains poorly understood. Here we aimed to fill gaps in the demographic and colonization history of the arctic fox by analyzing new ancient DNA data from fossil specimens aged from 50 to 1 thousand years from the Northern and Polar Urals, historic DNA from museum specimens from the Novaya Zemlya Archipelago and the Taymyr Peninsula and supplementing these data by previously published sequences of recent and extinct arctic foxes from other regions. This dataset was used for reconstruction of a time-calibrated phylogeny and a temporal haplotype network covering four time intervals: Late Pleistocene (ranging from 30 to 13 thousand years bp), Holocene (ranging from 4 to 1 thousand years bp), historical (approximately 150 years), and modern. Our results revealed that Late Pleistocene specimens showed no genetic similarity to either modern or historical specimens, thus supporting the earlier hypothesis on local extinction rather than habitat tracking.

## 1. Introduction

The Quaternary period is characterized by several alternating epochs of global warming (interstadials) and cooling (stadials) [1]. The last ice age (stadial) occurred between 114 and 11.7 thousand years ago (marine isotopic stages MIS 5a–d—MIS 2). Within it, climate fluctuations occurred with the coldest period (last glacial maximum–LGM) between 26.5 and 19 thousand years ago [2]. During the last stage, open and semi-open landscapes [3] and a mammoth faunal complex [4] were widespread in the territory of Northern Eurasia. The vegetation composition was dominated by cold-tolerant and drought-resistant species, and the fauna was dominated by cold-tolerant species of open landscapes (mammoth—*Mammuthus primigenius* Blumenbach, 1799; woolly rhinoceros—*Coelodonta antiquitatis* Blumenbach, 1799; horse—*Equus* Linnaeus, 1758; reindeer—*Rangifer tarandus* Linnaeus, 1758; steppe bison—*Bison priscus* Bojanus, 1825; musk ox—*Ovibos pallantis* H. Smith, 1827; collared lemming—*Dicrostonyx* Gloger, 1841; true lemming—*Lemmus* Link, 1795, etc.). The mammoth faunal complex included the arctic fox (*Vulpes lagopus* Linnaeus, 1758). In Asia, the southern border of its range was approximately 48° N [5]. About 11.7 thousand years ago, as a result of a sharp warming and humidification of the climate, a transition from the stadial (Late Pleistocene) to the interstadial (Holocene, MIS 1) occurred [6]. As a result, several key species of the mammoth complex became extinct: mammoth, woolly rhinoceros, and steppe bison [7]. The Early Holocene saw a rapid increase in forest area, leading to reductions in effective population sizes and fragmentation of the ranges of open-space species, including the arctic fox. Consequently, there was a reduction in the genetic diversity of many mammal species [8,9] and the formation of refugia [10]. This led to genetic drift, extinction, and a reduction in the effective number of populations. Anthropogenic impacts and interspecific competition, together with climate-induced changes, contribute to range contraction, which is more pronounced at the end of glacials.

The rapid development of DNA technologies and bioinformatics has facilitated the study of genomic data across time and space [11]. By utilizing the method of ancient DNA, it becomes possible to unveil genetic imprints of the climate impact on the populations of arctic and boreal fauna throughout the Late Quaternary period. Thanks to the exclusive fossil specimens, we are now able to explore the population dynamics during glacial and interglacial eras and, furthermore, evaluate how populations adapted to new environmental circumstances [12].

The arctic fox, *Vulpes lagopus* Linnaeus, 1758, is a well-known circumpolar species. The endangered Scandinavian population is of great interest to scientists for understanding the demographic history of *V. lagopus*. Despite the high level of interest, the population history of arctic foxes during the Late Pleistocene and Holocene remains poorly understood. Several studies of population structure have been inferred from microsatellite DNA [13,14] or fragmented mitochondrial DNA [15,16] of modern populations. Modern populations are genetically monomorphic in most areas, except for isolated regions such as the Commander Islands, which are not connected to the mainland by sea ice [17]. The ability to migrate long distances (about 90 km per day [18]) is a possible reason for the genetic similarity of different populations.

The last circumstance prevents discerning phylogeographic structure due to the extensive dispersal that occurs. In their groundbreaking study, Dalén et al. (2007) [19] utilized ancient DNA data obtained from European populations to reveal a captivating insight into the genetic diversity of arctic foxes. Their research has effectively proven that arctic foxes had considerably greater genetic diversity during the Late Pleistocene. Furthermore, the majority of fossil remains exhibit unique haplotypes, distinguishing them from present-day populations. Over the last decade, NGS technologies have been applied to examine ancient DNA [11], enabling Larsson et al. (2019) [20] to study fossil specimens from Belgium and Yakutia and reveal the lack of genetic inheritance among Late Pleistocene specimens in Europe. Thus, multiple studies have previously indicated the extinction of local populations that did not contribute to the genetic diversity observed in present-day individuals. Several theories on the origin of arctic foxes in Europe have been proposed [19]. According to the initial hypothesis, arctic foxes inhabited a vast area that emerged as a result of the melting ice caps by expanding their distribution. The second hypothesis suggests that arctic fox populations in Middle Europe became extinct at the onset of the Holocene. Later on, arctic foxes managed to colonize the northern regions. In this case, Eastern Russia may have been the source of the Scandinavian population, as suggested by Dalén et al. (2007) [19], but there was no direct evidence of genetic similarity between ancient specimens from Yakytia and modern samples from Norway, or between ancient specimens from Belgium and modern samples from northern regions. Thus, the sources of arctic foxes’ European expansion remain largely unknown.

Most recent studies have focused on European populations, whereas major parts of its past and current distribution remain unstudied genetically and thus the arctic fox population history is still poorly understood. Analysis of ancient DNA from additional Pleistocene and Holocene samples may fill the gaps.

The Ural Mountains are divided into regions along their North–South extent. The current distribution of the arctic fox is limited to the Polar and North Urals regions. However, in the past, during the Ice Age, *V. lagopus* inhabited the Middle and South Urals, and numerous well-preserved fossil remains of the species can be found throughout the Urals [21]. Climate warming, competition with the red fox, and hunting by ancient people cut down on the geographic range and genetic diversity in arctic fox populations. Deglaciation affected sea levels, causing boreal flora to shift northwards and greatly reducing the area of the arctic fox habitat. Furthermore, arctic foxes were hunted for their fur by Paleolithic people, and abundant fossils are located in ancient human sites. As for competition between foxes, *Vulpes vulpes* dominates *V. lagopus*. The eviction of arctic foxes from dens has been observed on several occasions [22]. The range of *V. vulpes* expanded northwards, causing arctic and red foxes’ geographic ranges to overlap [23,24].

The previously proposed hypotheses on ways of colonization of the Scandinavian Peninsula by arctic foxes may be verified by the study of unique fossil material from different regions of the Ural Mountains. Our research aimed to fill gaps in the demographic and colonization history of the arctic fox by (1) adding new fossil specimens aged from 50 to 1 kya, from the sites within the Northern and Polar Urals, (2) the inclusion of museum specimens of arctic foxes from the Novaya Zemlya Archipelago and the Taymyr Peninsula, and (3) adding to our data previously published sequences from other regions. With this approach, we can perform a comprehensive evaluation of population dynamics, both on a wide and local geographic scale with a particular attention to the region of the Ural Mountains.

## 2. Materials and Methods

### 2.1. Sampling

**Figure 1:**
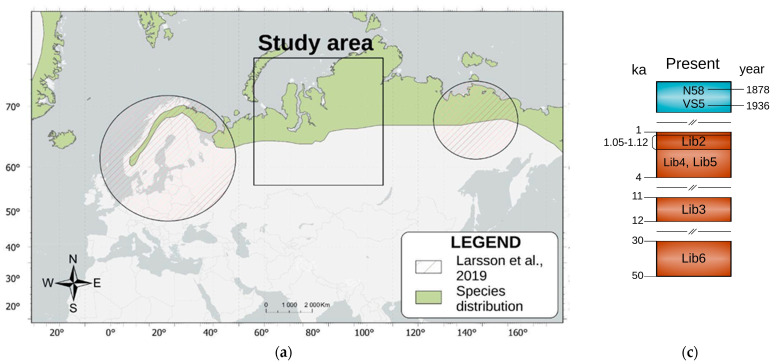

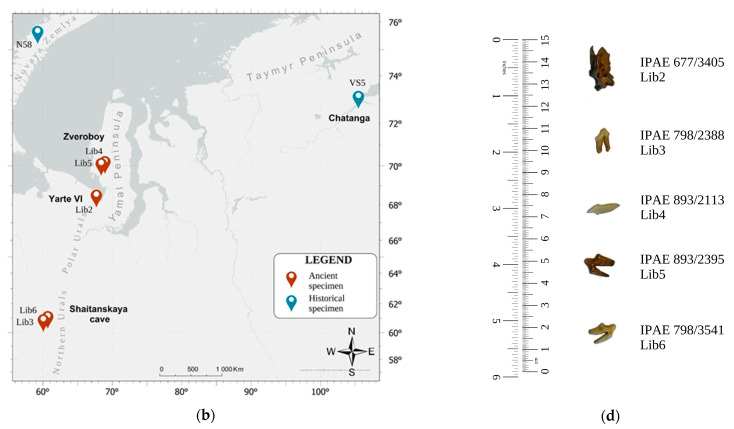
*Vulpes lagopus* sampling information. (**a**) The present-day arctic fox distribution (green color, IUCN Red List of Threatened Species. Version 2022-2. https://www.iucnredlist.org accessed 11 November 2023) Previously researched areas [20] indicated with red hatching lines. (**b**) Geographic location of ancient (red) and historical (blue) samples used in this study. (**c**) Time scale indicating the age of samples. (**d**) Arctic fox fossils used in this study.

The research is focused on five fossil specimens of *V. lagopus* collected from the Polar and Northern Urals (refer to Table 1 and Figure 1) from the collection of the Institute of Plant and Animal Ecology of the Russian Academy of Sciences. Two teeth from the deposits of the grotto Zveroboy in the Polar Urals were aged between four and one thousand years old according to radiocarbon dating [25]. *Os petrosum* was found in the settlement Yarte 6, the Polar Urals, the cultural layer of which is dated by the dendrochronological method at 1130–1050 years before present (kya bp) [26]. Two teeth samples were taken from the Shaitan Cave, the Northern Urals. One tooth originates from a layer dated by the radiocarbon method at 12–11 kya bp and the other is from a layer also dated using the radiocarbon method at 50–30 kya bp [27].

In addition, we also used two museum dry skin specimens of *V. lagopus* from the Novaya Zemlya archipelago and the Taymyr Peninsula. These specimens (dated 1878 and 1936) were obtained from the collection of the Zoological Institute of the Russian Academy of Sciences in Saint-Petersburg. For comparison, we took data from Larsson et al. (2019) [20]. The total sample included 59 *Vulpes lagopus* individuals: 31 modern, 18 historic (approximately 100–150 years), and 10 ancient (50–1 kya).

### 2.2. DNA Extraction, Library Preparation, and Sequencing

Ancient DNA from fossil remains was extracted in clean laboratory facilities at the National Research Center “Kurchatov Institute”, Russia. The preparation stage of DNA extraction was ultraviolet exposure for 10 min, removing the external specimen layer. After that, we used a tooth drill for bone milling. DNA extraction was performed using a silica-based method proposed by [28] and modified by [29,30,31]. To summarize, we incubated ca. 500 μg of bone powder with 2.5 mL of extraction buffer (described in [30]) at 42 °C for 48 h. Then, we transferred supernatant to a new tube, added 80 μg of silica pellets and 10 mL of binding buffer as described by [28]. After shaking and incubation for 3 h at room temperature, tubes were spun, and supernatants were removed and resuspended with 1 mL of washing buffer, consisting of 250 μg of 37% HCl and 50 mL of binding buffer. Solutions were spun for 2 min at 12,000 rpm, washed twice with 80% ethanol, resuspended with 80 μg of elution buffer (QIAGEN), and stored at +4 °C. The Qubit 4.0 Fluorometer (Thermo Fisher Scientific, Waltham, MA, USA) was used for measurement of DNA concentrations. Genomic libraries were made using the Ovation^®^ Ultralow Library System V2 kit (Tecan Group Ltd., Männedorf, Switzerland). Library quality was evaluated using the 2100 Bioanalyzer (Agilent Technologies, Santa Clara, CA, USA). Illumina Novaseq 6000 (Illumina, San-Diego, CA, USA) was used for pair-end (2 × 150 bp) whole-genome sequencing.

DNA from museum specimens was extracted in the laboratory of evolutionary genomics and paleogenomics of the Zoological Institute RAS in St. Petersburg using phenol-chloroform method according to protocols [32,33]. The N58 sample’s genomic library was prepared and sequenced at the National Research Center “Kurchatov Institute”, while the Evrogen Joint Stock Company, Russia, carried out the sequencing for the other VS5 sample.

### 2.3. Raw Data Analysis and Mitochondrial Genome Assembly

Firstly, we analyzed the raw data using FastQC software (Andrews, 2010 [34]). The initial stage of sequence processing includes cutting low-quality sequences (below 25 on the Phred-scale), overrepresented sequences, and Illumina adapters using Trimmomatic software [35]. Testing for damage patterns in ancient DNA sequences (postmortem damage such as hydrolytic deamination of cytosine and adenine at 5′-ends and 3′-ends of DNA strand, respectively) was carried out with DamageProfiler software [36]. We assembled mitochondrial genomes by mapping reads to the *Vulpes lagopus* reference mitochondrial genome (NCBI accession number NC_026529) using BWA MEM [37] and Bowtie2 [38]. The total number of reads is a result of summarization after two approaches. Additional steps such as filtering and duplicate removal were provided with Samtools software [39]. We analyzed all reads using BLAST [40] via Geneious Prime V. 2019.2.1 [41] to search erroneously mapping reads that were deleted. The minimum reference coverage of at least three was used to generate consensus sequences. Genome coverage was evaluated using the plotCoverage software implemented in deepTools2 [42]. Generating consensus sequences and multiple alignment (the Geneious multiple alignment algorithm) were realized in Geneious Prime V. 2019.2.1.

### 2.4. Metagenomic Analysis

Metagenomic analysis is used to evaluate the amount of endogenous DNA, besides surveying microbial diversity in samples. We used Kraken 2 software [43] for determining the taxonomic rank of the raw reads. We also exploited Krona [44] and Pavian [45] for visualization and comparison among all samples. Read length after trimming varied between 52 and 150 bp, leading to incorrect assignment of reads obtained from particular species. For further analysis, we consider all reads mapped to Canidae, not only *Vulpes lagopus*, as endogenous DNA. Therefore, to count the amount of endogenous DNA, we used reads which were defined as Canids.

### 2.5. Phylogenetic Analysis

The search for a suitable substitution model was performed using PartitionFinder. Complete mitochondrial DNA was partitioned into 13 protein coding genes, rRNAs, tRNAs, and D-loop. We also took into account codon positions for protein coding genes. We reconstructed a time-calibrated phylogeny using Beast V.2.7.4. [46] for separate subsets using different substitution models, e.g., GTR + I, GTR + G, GTR + I+G, and HKY + I (see Table S3).

We used a strict clock model with default parameters. Tip dates were specified for all samples. To obtain the Bayesian phylogeny, two Markov chain Monte Carlo analyses were run for 100 million generations each and then merged using logCombiner (v2.7.4 included in the BEAST package), discarding the first 10% of generations as burn-in. The effective sample size of parameters was estimated to check the quality of the analysis using Tracer (v2.7.4, included in the BEAST package). The resulting tree was calculated using treeAnnotaitor and visualised using FigTree version 1.4.4 (http://tree.bio.ed.ac.uk/software/figtree/, accessed on 12 October 2023).

Furthermore, we constructed a temporal haplotype network according to the TempNet script [47] using R V.4.2.3 software (R Core Team, 2022, Vienna, Austria). Our dataset was divided into four groups representing different time intervals: Pleistocene (ranging from 30 to 13 thousand years bp), Holocene (ranging from 4 to 1 thousand years bp), historical (approximately 150 years), and modern. We created the reduced dataset by removing any ambiguities in the alignment for haplotype generation. This dataset excluded two Late Pleistocene specimens from the North Urals (Lib3, Lib6), one Holocene specimen from the Polar Urals (Lib5), and also the outgroup. The total number of sequences was 56 with a length of 3044 bp.

## 3. Results

We assembled six complete and fragmented mitochondrial genomes of *Vulpes lagopus* and one of *V. vulpes*. Mitochondrial genomes of two ancient specimens from the Polar Urals (Zveroboy, Yarte VI; 4–1 kya) and one historic from Taymyr Peninsula were complete. The other mitochondrial genomes of the arctic fox (Shaitanskaya cave, 50–30 and 10 kya; the Novaya Zemlya Archipelago) and the red fox (Zveroboy, 4–1 kya) were partial. DNA extracted from fossil remains contains both endogenous and exogenous, non-species of interest genome fragments. Traditionally, the amount of endogenous DNA is estimated at below 1% [48]. In our case, the percentage of endogenous ancient DNA varied from 2 to 11 in four out of five specimens.

### 3.1. Raw Data

The total number of raw reads varies from 69.3 to 168.8 million with the percentage of duplicates ranging from 49.9 to 93 for ancient samples (Table S2). An average number of raw reads for museum specimens was an order of magnitude lower (Table S2). A Phred quality score was above 30 and the minimal read length was 52 after trimming. An average read length after trimming is represented in Table 2. The assembly quality of the ancient specimens is shown in Figure S1. Three of five datasets did not contain enough information for correct damage estimation. For the other two samples, no damage was found, so no further read cutting was carried out.

### 3.2. Metagenomic Analysis

Table S1 represents extracted DNA concentrations. DNA concentrations from fossil remains varied between 0.114 and 0.817 ng/µL except Lib2 with a concentration of 9.92. Despite such unexpected high values, further computational analyses did not detect any contamination. The main results of the metagenomic analysis are shown in Table S4; additionally, we present pie charts for all specimens for clarity (Figure S3). We calculated the percentage of classified and unclassified reads, which represent the fraction of sequences having homologous sequences in GenBank NCBI. The percentage of classified reads varied from 47.5 to 61.8 for ancient specimens.

Historic samples have higher values of 86.7 and 94.6%. Ancient samples have numerous microbial reads; otherwise, historic samples have a high value of chordate reads. The value of chordate reads varies from 3.43 to 16.1% for ancient samples. The minimal value of endogenous DNA, 0.14% (Canidae reads), belongs to the ancient sample dated to about 50–30 kya (Lib6); the Holocene sample (Lib4) endogenous DNA accounts for 11.90%. Two museum samples have the highest percentage of endogenous DNA, 43.3% for the sample from the Novaya Zemlya archipelago and 84.5% from the Taymyr Peninsula.

### 3.3. Mitochondrial Genome Assembly

The main results of the arctic fox’s mitochondrial genome assembly are presented in Table 2. We obtained three complete mitochondrial genomes of two ancient (Lib2, 4: 4–1 kya) and one historic sample. We also received four fragmented mitochondrial genomes for other samples. The size of the mitochondrial genome is the shortest for Late Pleistocene samples (Lib3, 6).

We estimated the reference genome coverage of ancient samples (Figure S2). The coverage of Late Pleistocene samples, especially Lib3, was very poor. One Holocene sample (Lib5), despite its age, also turned out to be poorly covered. The mitochondrial genome of two Holocene samples (Lib2, 4) were mostly covered by 20× to 30× reads.

### 3.4. Phylogenetic Analysis

**Figure 2:**
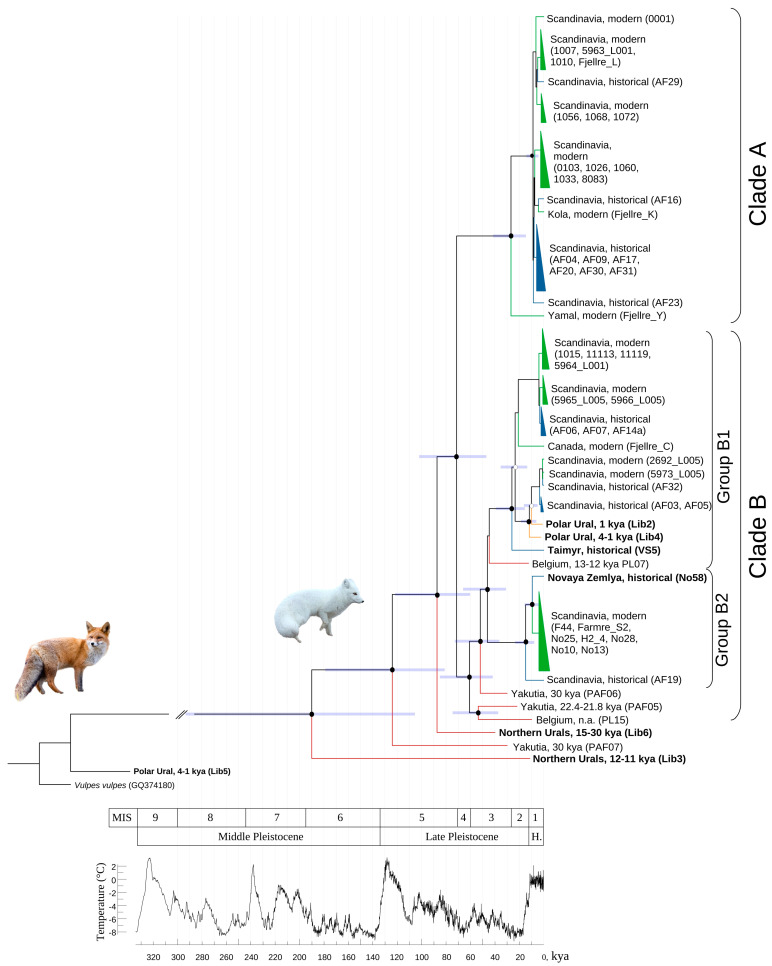
*Vulpes lagopus* phylogeny reconstruction with divergence dating inferred in BEASTv. 1.10 software using *Vulpes vulpes* as an outgroup. Nodal support in Bayeasian posterior probabilities (BPP) are indicated by colors (>0.99 is black, >0.95 is white, <0.95 not shown). Confidence intervals for the age of the nodes are represented by 95% HPD (purple bars). MIS intervals for Pleistocene and Holocene corresponds to the temperature fluctuation chart (modified from Petit et al., 1999 [49]). Foxes’ photographs were taken from iNaturalist. Available from https://www.inaturalist.org. Accessed [24 October 2023]. The colors designate samples of arctic foxes of different age: green—modern, blue—historical, yellow—ancient.

The final dataset of 16,801 bp sequences included 60 specimens; among these were 59 *Vulpes lagopus* individuals and one *Vulpes vulpes* as an outgroup. The phylogenetic tree with 95% HPD (height posterior density) was constructed using the Bayesian approach, shown in Figure 2. The results of the phylogenetic analysis definitely showed that the one Holocene sample (Lib5) belongs to a red fox.

According to the phylogenetic tree, the divergence of the arctic fox in the Palearctic started approximately 190 kya (190.012 kya; highest posterior density = 105.521–239.264 kya). Within all samples studied, three Late Pleistocene specimens (Lib3, PAF07, Lib6) are early derivatives. Basically, modern and historical specimens form two main clades (A and B), which split around 70 kya (71.407 kya; highest posterior density = 47.012–101.249 kya). All studied earlier samples from Scandinavia [18], both modern and historical, found to be within these two clades. However, clade A includes a modern specimen from the Kola Peninsula and one from the Yamal Peninsula; clade B includes a modern specimen from Canada and historical specimens from the Taymyr Peninsula and the Novaya Zemlya Archipelago. Three Late Pleistocene specimens (PAF05, PAF06, PL15) are sister to clade B, and two of them form a separate clade (PAF06, PL15). Two separate groups within clade B (B1 and B2) split around 50 kya (46.208 kya; highest posterior density = 30.947–66.082 kya). The Late Pleistocene Belgian specimen (PL07) was sister to B1, and two Holocene specimens (Lib2, 4) were within the group B1.

**Figure 3:**
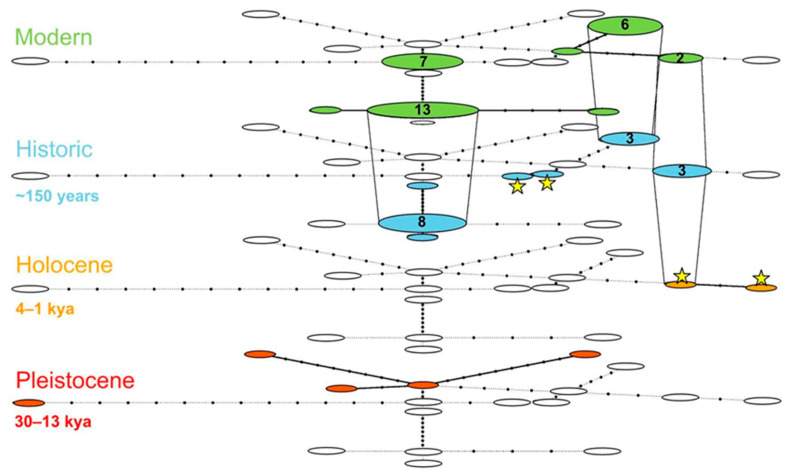
Late Pleistocene samples aged 30–13 thousand years are designated by red, Holocene samples aged 4–1 thousand years by orange. Blue corresponds to historical samples around 150 years old, and green to modern samples. Uncolored ovals mark missing haplotypes. Haplotypes occurring through several periods are connected by vertical lines. The numbers within the circles indicate the number of samples for each haplotype if there is more than one. The dots indicate the number of nucleotide substitutions between haplotypes. The samples analyzed in the current study are marked with a star.

To estimate the temporal and spatial haplotype diversity, we built the temporal haplotype network divided into the following epochs: Pleistocene, Holocene, historic, and modern (Figure 3). We excluded three samples (Lib5, Lib3, and Lib6) from the Urals because their presence reduced haplotypic diversity. The analyzed fragment length 3044 bp of mitochondrion contained 49 variable positions. The total number of haplotypes was 17 for 56 sequences. All Pleistocene samples (5) were represented by unique haplotypes. None of them has survived through the ages. Two samples from the Holocene belong to different haplotypes. One of them survived through the 20th century until now. Among the historical samples, seven haplotypes were distinguished; within them, one has survived from the Holocene to the recent past. Three haplotypes found in historical samples survived to the present, and four haplotypes are unique to historic samples. One haplotype was dominant both for historical and modern periods. Additionally, there are seven haplotypes in the modern period, two of them being inherited from the historical period and one survived from the Holocene. The remaining four modern haplotypes are unique for contemporaneity.

## 4. Discussion

### 4.1. Late Pleistocene Climate Oscillations as a Main Trigger of Diversification in the Arctic Fox

Climate change has shifted the borders of tundra, forest-tundra, and taiga zones, which therefore determine the geographic range of the arctic fauna, including the arctic fox. The earliest arctic fox fossil record in Europe dates back to ca. 200 kya [50], when arctic foxes apparently spread across Eurasia. This is consistent with our divergence time estimation (Figure 2). According to our data, the first split of the *Vulpes lagopus* clade began ca. 190 kya, coinciding with the beginning of the Penultimate Glacial Period, lasting from 195 to 135 kya (MIS 6 [51]). During this period, a vast ice sheet exceeding the size of even the glaciers during the LGM emerged [52]. The following epoch was the Last Interglacial, which was observed during the MIS 5e (130–115 kya) and characterized by a warmer climate than the present day [53,54].

The Last Glacial Period occurred from ca. 115 to 11.7 kya. The early stages, MIS 5d–5a, are characterized by several alternating cooling and warming epochs. The MIS 5d and 5b substages, dated to ca. 115–92 and ca. 92–85 kya, respectively, are associated with the onset of glaciation in Norway’s mountains [55] and the Kola Peninsula. The arctic fox most likely began its post-interglacial expansion during these periods of the Last Glaciation. Three Late Pleistocene specimens (Lib3, Lib6, Paf07, Figure 2) from the North Urals and Siberia, splitting earlier than other groups, have the divergence time estimates corresponding to this period. Earlier, in the stage of MIS5a (85–71 kya), there was warming, which was replaced by increased cooling up to 70 kya, which is comparable to the age of the common ancestor of two modern clades (A and B, Figure 3).

The MIS3 (ca. 60–27 kya) stage is characterized by cold conditions. All over Eurasia, the prevailing landscape was represented by open tundra-steppe [56], favorable habitat for arctic foxes. The onset of this epoch is marked by the emergence of clade B, which includes two Late Pleistocene specimens (PAF05, PL15) from Belgium and Siberia that are basal to others in clade B (Figure 2) and represent a separate dead-end branch. The major diversification within clade B began around 58–53 kya when the maximum of Norway’s glaciation was reached [55]. Remarkably, two Holocene specimens (Lib2, 4) were found within one of the clade B groups; among these, one has the haplotype that has survived until the present (Figure 2). The clade A originated ca. 30 kya, 30 kya earlier than clade B. There were no Pleistocene or Holocene samples grouped with clade A, and we cannot determine any ancestors or its origin. In terms of the Pleistocene specimens, none of the seven survived into the Holocene and are not considered ancestral.

The most severe conditions occurred during the LGM (27–19 kya). Arctic foxes’ habitats were most extensive, reaching the southern limit of the tundra-steppe. The rapid diversification occurred simultaneously ca. 25 kya in both clades of *V. lagopus*, in spite of the different times of its origin. The extreme environmental conditions did not persist throughout the entire Eamian glaciation, but only during the LGM. The emergence of two main modern clades in the phylogenetic tree provides a clear illustration, perfectly supporting the idea that the arctic fox significantly dispersed roughly 25,000 years ago during the onset of the late pleniglacial period.

The Last Glacial Period came to an end ca. 18 kya, marked by a swift deglaciation process. The Bölling/Alleröd warming (14.7–12.7 kya) saw the revival of forests in Europe. However, during the subsequent younger Dryas period (12.7–11.7 kya), a cooling phase occurred, leading to the disappearance of forests in northern Central Europe [56]. The annual temperature was higher in the Middle Holocene than today. Conditions during the LGM were cold but stable, in contrast to the Early Holocene. The rapid climate change during this period had profound consequences, resulting in genetic turnover among different species [57] and our data on ancient and historical samples, despite small numbers, also indicate the continuous decrease in genetic diversity and genetic turnover in arctic foxes through these time periods.

### 4.2. Habitat Tracking or Local Extinctions?

Abrupt changes in climatic conditions can cause a significant decrease in genetic diversity [58], which occurs due to local extinctions and habitat tracking (e.g., [59]). These hypotheses explore how species respond to climate change. Previously published research suggests the local extinction of arctic fox populations in Europe [19] was a result of climate change.

Apart from the arctic fox, there are other fascinating members of the arctic fauna that also deserve attention when it comes to unraveling the captivating global events that transpired at the turn of the Pleistocene and Holocene. The case of the arctic fox’s prey, *Lemmus lemmus* Linnaeus, 1758, provides undeniable evidence of the significant difference in genetic diversity between past populations and the present ones that lack any ancestral inheritance [60]. Similar results indicating population extinctions and genetic turnover over a large geographic scale within the Late Pleistocene were obtained in the paleogenomic study of another specialized arctic rodent, also the main prey of the arctic fox, the collared lemming, *Dicrostonyx torquatus* Pallas, 1778 [61]. The authors convincingly demonstrated that lineage replacements were geographically widespread, indicating a pattern of repeated population extinctions across much of the collared lemming’s Eurasian distribution. It is noteworthy that this new paleogenomic data refuted earlier findings on population continuity in collared lemmings in the Northern Urals over 25 kya [62]. Our results reported here rather speak in favor of local extinction of the arctic fox in the Urals at the end of the LGM, although this conclusion needs to be confirmed by the study of additional ancient DNA samples.

Habitat tracking is the alternative hypothesis explaining how species respond to changing conditions. The shift in plant zones has opened up new territories and habitats for migrating species. This event showcases the genetic inheritance of the willow (*Lagopus lagopus* Linnaeus, 1758) and rock (*Lagopus muta* Montin, 1781) ptarmigan in Europe and Siberia over a span of 20 thousand years [63].

### 4.3. Where Do the Arctic Foxes of the Modern European North Come from?

The phylogenetic tree (Figure 2) provides a fascinating insight into how the modern Scandinavian population could have emerged from a plural resettlement process, giving rise to two distinct clades. Until recently, it has been questioned whether the Beringian populations might be the ancestors of modern Scandinavian foxes. According to multiple studies, Beringian populations were the source of postglacial expansion for different species both in the westward and eastward directions. For instance, by conducting a phylogeographic analysis using fragments of wolverine (*Gulo gulo* Linnaeus, 1758) mtDNA, the hypothesis of the Beringian origin of recent populations was proposed [64]. Recent modern and ancient DNA research on gray wolves (*Canis lupus* Linnaeus, 1758) suggests the colonization of western Eurasia by wolf populations from east Eurasia [65]. The addition of new data from unexplored areas and times sheds light on our understanding of the population history of arctic foxes. Further genomic and paleogenomic studies, based on scarce resources, have fuelled speculation about the origin of Scandinavian populations. Notwithstanding the lack of raw data, Larsson et al. (2019) [20] hypothesized a Beringian origin for modern Scandinavian populations. We intend to provide compelling evidence for the genetic connection between arctic fox populations in Norway and the Urals.

Based on the formation of two distinct clades, we can propose that Scandinavian settlement occurred on two separate occasions, originating from different population sources following deglaciation. However, the Late Pleistocene specimens that have now been studied from Europe (two specimens), Yakutia (three specimens), and the North Urals (two specimens) are not closely related to any of the two modern clades.

The modern arctic foxes found in the Scandinavian Peninsula can trace their genetic lineage back to the ancient arctic foxes that roamed the Polar Urals, dated to 4 to 1 thousand years ago. This connection is facilitated by a shared Holocene haplotype, which is present in both present-day and historical specimens. As for clade A, it is challenging to devise any hypotheses regarding its formation during the Ice Age.

Our research did not uncover any genetic continuity between arctic foxes from the Northern Urals (dated 50–30 kya) and arctic foxes from the Polar Urals (dated 4–1 kya). This finding aligns with the prevailing theories of local extinction during the transition from the Pleistocene to the Holocene, reflecting the impact of climate change. During the deglaciation process after the LGM, the boreal vegetation zone noticeably shifted towards the north, unavoidably impacting the flora and fauna.

Meanwhile, the Holocene arctic foxes and the modern arctic fox from Yamal belong to distinct clades, indicating that the Yamal population has originated recently from different sources. The evolutionary origin of modern populations of arctic foxes on Yamal is extraneous to the populations of arctic foxes that inhabited these areas earlier. Nevertheless, it is crucial to emphasize the importance of conducting further research of additional specimens in order to acquire more precise and reliable data.

## 5. Conclusions

In our study, we examined unique fossil material represented by teeth, bone, and skin remains of arctic foxes. A distinctive feature of the data used for analysis is the presence of data points from the same site, specifically from the Urals region, across consecutive time slices such as the Late Pleistocene and the Holocene. Our expectations about arctic foxes’ genetic inheritance throughout the Urals during the last fifty thousand years were not met. Based on our data, it appears that local extinction is the more likely scenario, which has also been proposed for the European region. Moreover, our data contradict previously assumed hypotheses on the arctic fox’s colonization of Scandinavia from Beringian populations [19], and suggest another scenario, supposing the Urals region as the source for westward colonization. The most probable period is the Holocene instead of the Late Pleistocene. Thereby, none of the Late Pleistocene specimens exhibit any genetic similarity to either modern or museum specimens, as further demonstrated by the haplotype network. Unfortunately, in line with current data, we are unable to establish any phylogenetic inheritance between the Late Pleistocene and either historic or modern individuals.

## Figures and Tables

**Table 1:** *Vulpes lagopus* specimens.

Specimen_ID	Location	Type of Material	Latitude	Longitude	Age	Accession №
IPAE_798/3541	Shaitanskaya cave, the Northern Urals	P3/	60.42 N	60.22 E	50–30 kya	OR880607
IPAE_798/2388	Shaitanskaya cave, the Northern Urals	p/2	60.42 N	60.22 E	12–11 kya	OR880605
IPAE_893/2113	Zveroboy, the Polar Urals	i/3	67.7 N	67.85 E	4–1 kya	OR880606
IPAE_893/2395	Zveroboy, the Polar Urals	p/4	67.7 N	67.85 E	4–1 kya	OR880610
IPAE 677/3405	Yarte VI, the Polar Urals	*os petrosum*	68.54 N	69.57 E	1130–1050 years ago	OR880604
ZIN_5877	the Novaya Zemlya archipelago	tanned skin	74.54 N	57.63 E	1878 year	OR880608
ZIN_6079	Chatanga (the Taymyr Peninsula)	tanned skin	71.96 N	102.40 E	1936 year	OR880609

Institute acronyms: IPAE—the Institute of Plant and Animal Ecology of RAS. ZIN—the Zoological Institute of RAS.

**Table 2:** Mitochondrial genome characteristics.

Age	Name	Nucleotide Composition, %				GC Count, %	Total Number of Raw Reads	Total Number of Filtered Reads	Length, bp
		T	A	G	C				
1130–1050 years ago	Lib2	27.8	31.3	14.7	26.2	40,9	5066	3769	16,733
12–11 kya	Lib3	26.3	31.5	14.7	274	42.2	1964	140	1523
4–1 kya	Lib4	27.8	31.3	14.7	26.2	40.9	19,190	4395	16,758
4–1 kya	Lib5	27.8	31.9	15.4	24.9	40.3	8015	753	9205
50–30 kya	Lib6	28.7	30.2	15.2	25.8	41.1	681	334	5809
1878 year	N58	28.0	31.5	14.8	25.7	40.5	941	829	12,427
1936 year	VS5	27.8	31.3	14.7	26.2	40.9	64,285	22,966	16,816

## Data Availability

Partial mitochondrial genomes of *Vulpes lagopus* and *V. vulpes* obtained in this study were submitted to the NCBI GenBank database under the accession numbers OR880604–OR880610. Also, FASTA files and annotations of sequences are available from https://github.com/ZaTaxon/Vulpes_lagopus_aDNA accessed on 11 November 2023.

## Supplementary Materials

The following supporting information can be downloaded at: https://www.mdpi.com/article/10.3390/biology12121517/s1, Figure S1: Ancient DNA damage patterns (Lib2–6); Figure S2: Ancient samples reference genome mapping coverage (Lib2–Lib6); Figure S3: Pie charts illustrate results of metagenomic analysis for all specimens; Figure S4: Phylogenetic tree without labels compression; Table S1: Total DNA concentration; Table S2: Sequencing data; Table S3: Substitution models; Table S4: Metagenomic analysis results of ancient and museum samples of arctic fox.
