# Supplementary material for: In Search of the Elusive North: Evolutionary History of the Arctic Fox (Vulpes lagopus) in the Palearctic from the Late Pleistocene to the Recent Inferred from Mitogenomic Data
Source: Biology (Basel). 2023 Dec 12;12(12):1517. doi: 10.3390/biology12121517 (PMC10740874; doi:10.3390/biology12121517)
Supplement: Supplementary file 1 [file biology-12-01517-s001.zip › biology-2745782-supplementary.pdf]

Table S1. Total DNA concentration.

Sample	DNA Concentration (ng/µl)
Lib2	9.92
Lib3	0.114
Lib4	0.301
Lib5	0.817
Lib6	0.451
N58	20.9
VS5	>100

Table S2. Sequencing data.

ID	Number of Raw Reads, MM	% Duplicates	Average Reads Length after Trimming, bp
Lib2	168.8	49.9	125
Lib3	69.3	93.0	133
Lib4	101.7	78.6	129
Lib5	69.7	90.0	124
Lib6	117.0	59.0	128
N58	2.9	9.6	124
VS5	14.9	15.0	144

Table S3. Substitution models.

Group	Model	Number of Sites	Partitions
1	GTR+I	7722	pos1_COX2, pos1_CYTB, tRNA-Arg, tRNA-Glu, tRNA-Ile tRNA-Leu, pos1_COX3, tRNA-Pro, pos1_ND3, pos1_ND4l tRNA-Ser, tRNA-Leu, tRNA-Met, tRNA-Cys, rRNA_12S,

			tRNA-Val, pos1_ATP6, tRNA-Ser, tRNA-Asn, pos1_ND1, pos1_ND2, tRNA-Tyr, pos1_COX1, pos1_ND6, rRNA_16S tRNA-Lys, pos1_ND5, tRNA-Phe, pos1_ATP8, pos1_ND4 tRNA-Thr, tRNA-His, tRNA-Ala, tRNA-Tyr
2	HKY+I	3940	pos2_ND2, tRNA-Gly, tRNA-Asp, pos2_CYTB, pos2_COX pos2_ND5, pos2_ATP6, pos2_ND3, pos2_ND4, tRNA-Gln pos2_COX2, pos2_ND1, pos2_ND4L, pos2_ND6, pos2_CO
3	GTR+G	3800	pos3_COX2, pos3_ND2, pos3_COX1, pos3_ATP8, pos3_COX3, pos3_ND5, pos3_ND6, pos3_ND1, pos3_ND4 pos3_CYTB, pos3_ND4L, pos3_ATP6, pos3_ND3
4	GTR+I+G	1339	D-loop, pos2_ATP8

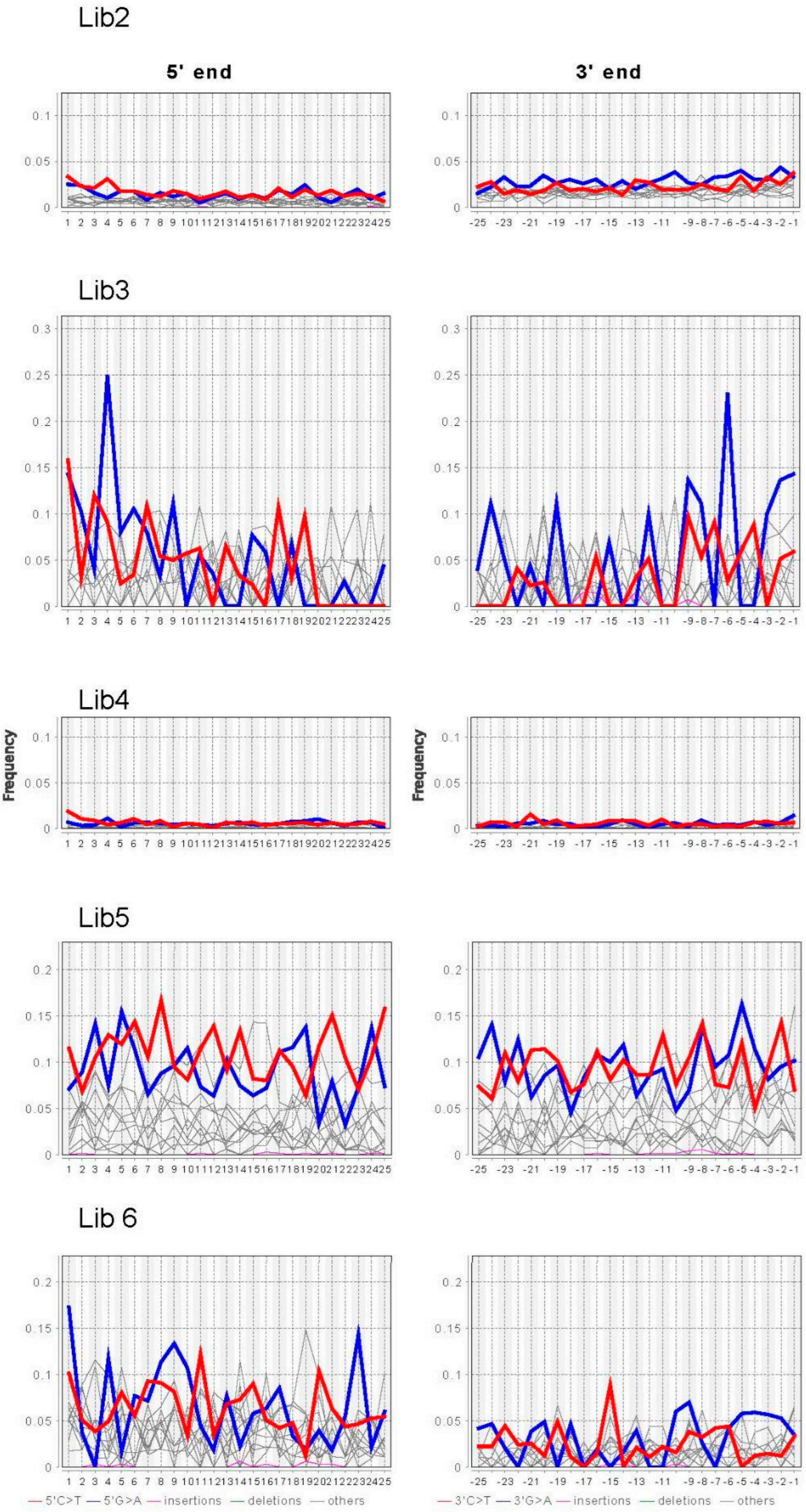
Lib2
5' end
3' end
Lib3
Lib4
Frequency
Lib5
Lib 6
5'C>T
5'G>A
3'C>T
3'G>A
insertions
deletions
others

Figure S1. Ancient DNA damage patterns (Lib2–6). The nucleotide positions of the 5' and 3' ends of the reads are shown on the horizontal axis, with the proportion of incorrectly inserted bases represented on the vertical axis. The red and blue colors indicate cytosine-to-thymine substitution and guanine-to-adenine substitution, respectively. Insertions are shown in pink, deletions in green.

Table S4. Metagenomic analysis results of ancient and museum samples of arctic fox.

LAB_ID	Number of Raw Reads	Classified Reads [1]	Unclassified Reads	Microbial Reads	Chordate Reads	Canidae Reads
Lib2	127,039,521	61.8%	38.2%	56%	5.36%	2.21% (2,805,236)
Lib3	53,124,906	48.5%	51.5%	35.3%	11.9%	3.64% (1,933,909)
Lib4	75,597,943	60.1%	39.9%	43.6%	16.1%	11.90% (8,992,487)
Lib5	43,505,102	47.5%	52.5%	35.3%	11.5%	6.11% (2,659,135)
Lib6	85,793,479	57.6%	42.4%	53.8%	3.43%	0.14% (116,518)
N58	2,301,319	86.7%	13.3%	29.9%	55.2%	43.3% (997,317)
VS5	7,483,996	94.6%	5.37%	5.1%	89.4%	84.5% (6,328,500)

[1] The percentage in all columns is specified from the total number of reads.

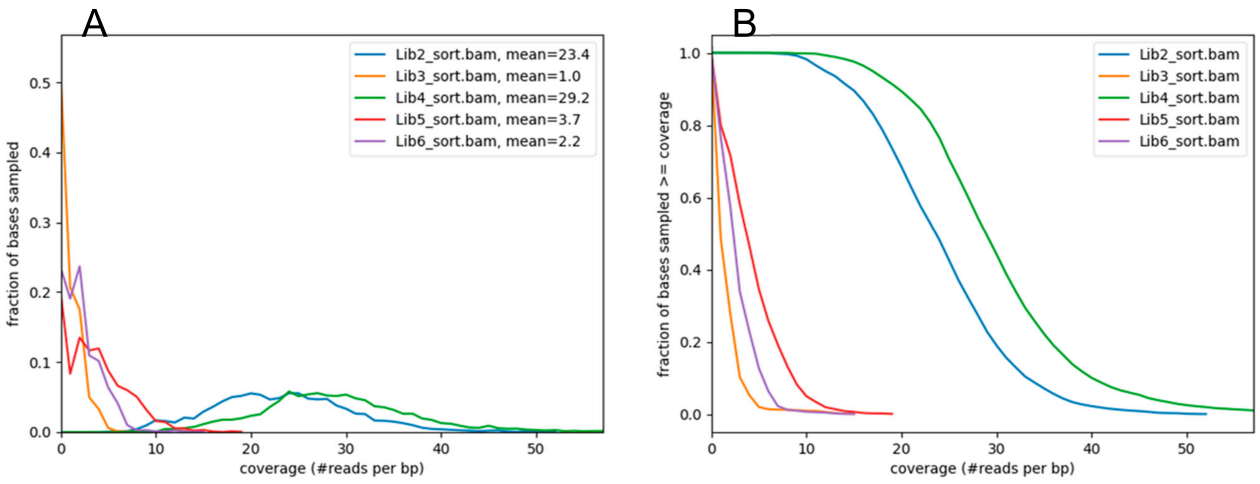

Figure S2. Ancient samples reference genome mapping coverage (Lib2–Lib6). (A) Plot represents the frequencies of the found read coverages and the mean coverage value in the upper right corner. (B) Plot represents the fraction of the genome that has a specified depth of sequencing.

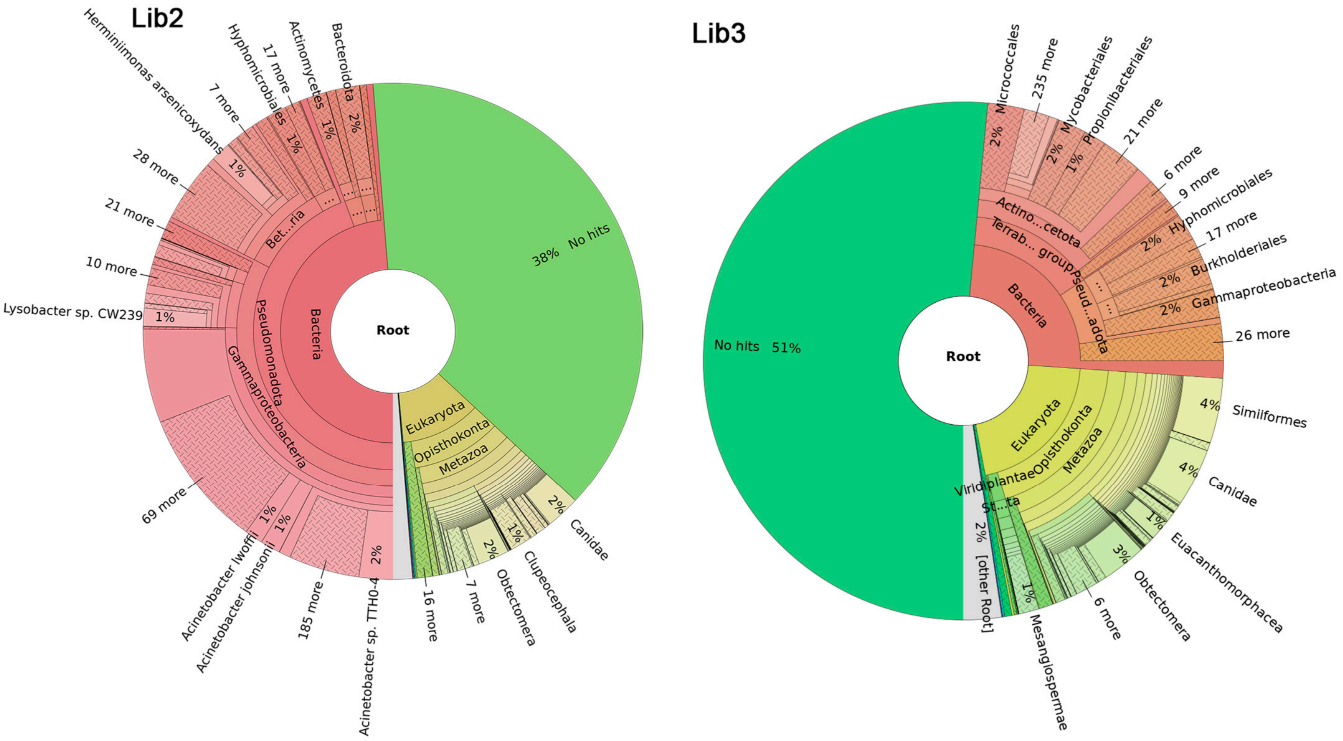
Lib2
Root
Bacteria
Pseudomonadota
Gammaproteobacteria
Bet...ria
No hits 38%
Eukaryota
Opisthokonta
Metazoa
Canidae 2%
Clupeocephala
Obtectomera 1%
7 more 2%
16 more
Acinetobacter sp. TTH0-4 2%
185 more
Acinetobacter johnsonii 1%
Acinetobacter lwoffii 1%
69 more
Lysobacter sp. CW239 1%
10 more
21 more
28 more
Herminiimonas arsenicoxydans 1%
7 more
Hyphomicrobiales 1%
17 more
Actinomycetes 1%
Bacteroidota 2%
Lib3
Root
No hits 51%
Bacteria
Actino...cetota
Terrab... group
Pseud...adota
Micrococcales 2%
235 more
Mycobacteriales 2%
Propionibacteriales 1%
21 more
6 more
9 more
Hyphomicrobiales 2%
17 more
Burkholderiales 2%
Gammaproteobacteria 2%
26 more
Eukaryota
Opisthokonta
Metazoa
Viridiplantae
St...ta
Siluriformes 4%
Canidae 4%
Euacanthomorphacea 1%
Obtectomera 3%
6 more
Mesangiospermae 1%
[other Root] 2%

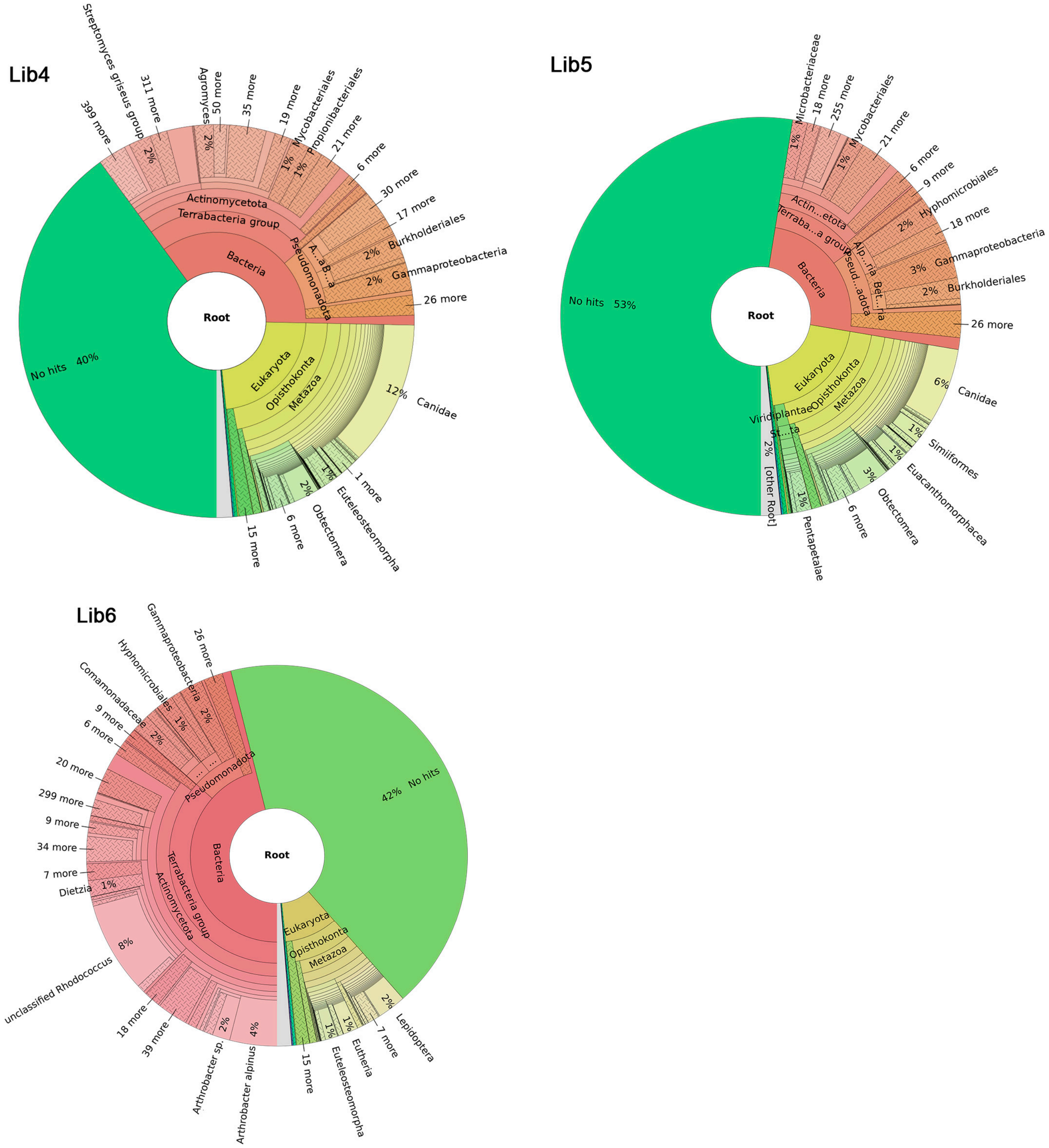

Lib4
Root
Bacteria
Terrabacteria group
Actinomycetota
Streptomyces griseus group
399 more
311 more
2%
Agromyces
50 more
2%
35 more
19 more
Mycobacteriales
1%
Propionibacteriales
1%
21 more
6 more
30 more
17 more
Burkholderiales
2%
Gammaproteobacteria
2%
26 more
Pseudomonadota
A...aB...a
No hits 40%
Eukaryota
Opisthokonta
Metazoa
12% Canidae
1 more
1%
Euteleosteomorpha
2%
Obtectomera
6 more
15 more
Lib5
Root
Bacteria
Terraba...a group
Actin...etota
Microbacteriaceae
1%
18 more
255 more
Mycobacteriales
1%
21 more
6 more
9 more
Hyphomicrobiales
2%
18 more
Gammaproteobacteria
3%
Burkholderiales
2%
26 more
Pseud...adota
Alp...ria
Bet...ria
No hits 53%
Eukaryota
Opisthokonta
Metazoa
Viridiplantae
St...ta
6% Canidae
1%
Simiiformes
1%
Euacanthomorphacea
3%
Obtectomera
6 more
1%
Pentapetalae
2% [other Root]
Lib6
Root
Bacteria
Terrabacteria group
Actinomycetota
Pseudomonadota
Gammaproteobacteria
26 more
2%
Hyphomicrobiales
1%
Comamonadaceae
2%
9 more
6 more
20 more
299 more
9 more
34 more
7 more
Dietzia 1%
unclassified Rhodococcus 8%
18 more
39 more
Arthrobacter sp. 2%
Arthrobacter alpinus 4%
42% No hits
Eukaryota
Opisthokonta
Metazoa
2%
Lepidoptera
7 more
1%
Eutheria
1%
Euteleosteomorpha
15 more

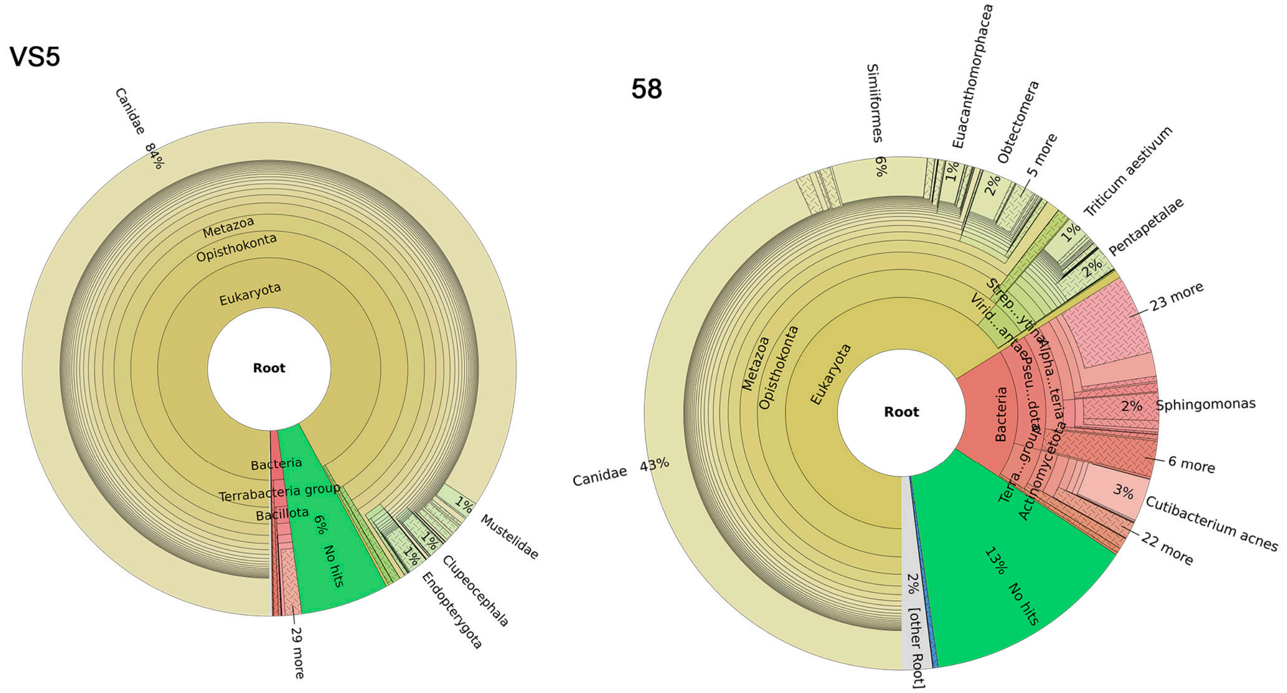

Figure S3. Pie charts illustrate results of metagenomic analysis for all specimens.

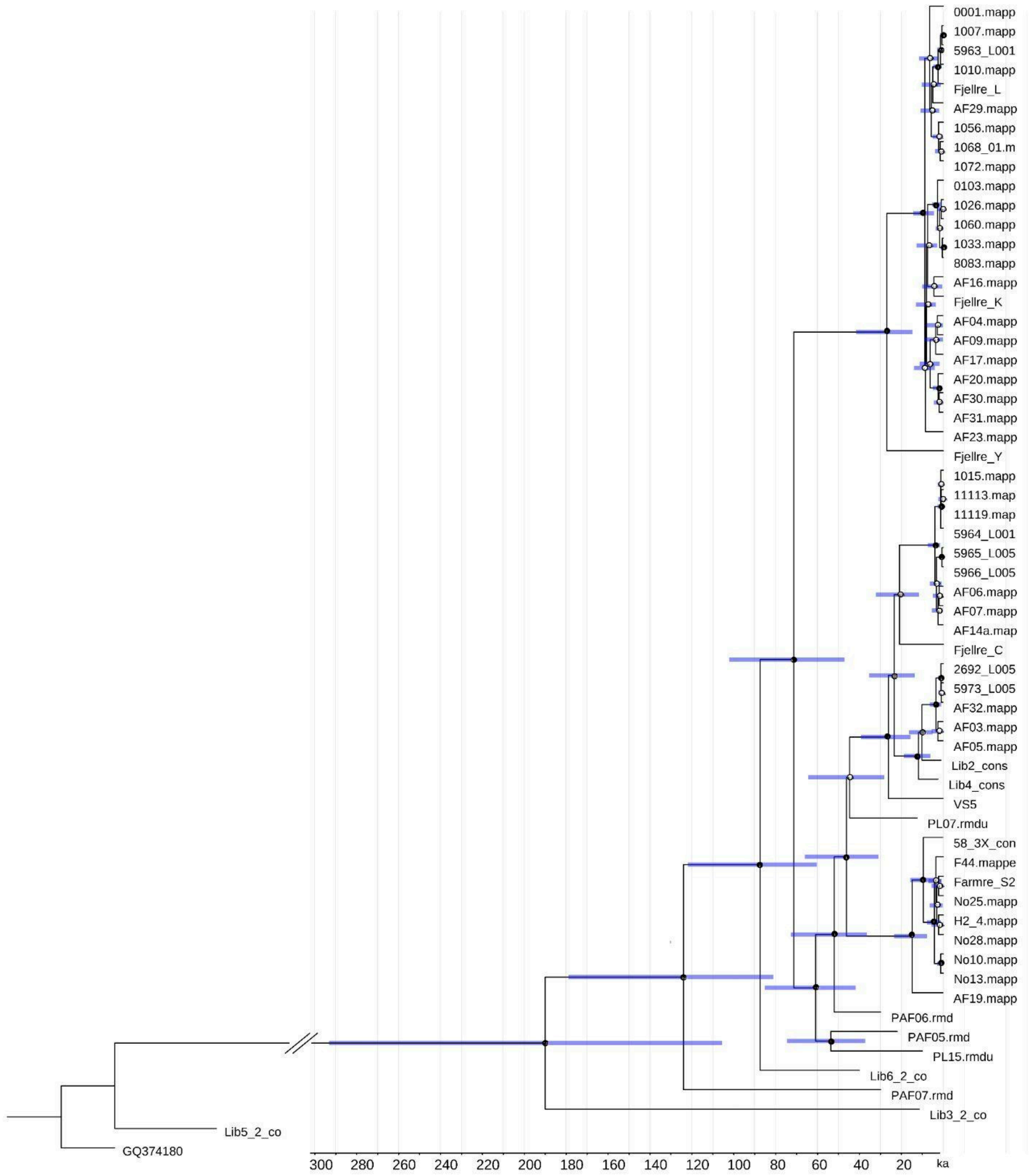

Figure S4. Phylogenetic tree without labels compression.
